# Abbreviated MR Protocols in Prostate MRI

**DOI:** 10.3390/life12040552

**Published:** 2022-04-07

**Authors:** Andreas M. Hötker, Hebert Alberto Vargas, Olivio F. Donati

**Affiliations:** 1 Institute of Diagnostic and Interventional Radiology, University Hospital Zurich, University of Zurich, 8091 Zurich, Switzerland; olivio.donati@usz.ch; 2 Memorial Sloan Kettering Cancer Center, Department of Radiology, New York, NY 10065, USA; vargasah@mskcc.org

**Keywords:** prostate cancer, magnetic resonance imaging, artificial intelligence, dynamic contrast-enhanced, diffusion-weighted imaging

## Abstract

Prostate MRI is an integral part of the clinical work-up in biopsy-naïve patients with suspected prostate cancer, and its use has been increasing steadily over the last years. To further its general availability and the number of men benefitting from it and to reduce the costs associated with MR, several approaches have been developed to shorten examination times, e.g., by focusing on sequences that provide the most useful information, employing new technological achievements, or improving the workflow in the MR suite. This review highlights these approaches; discusses their implications, advantages, and disadvantages; and serves as a starting point whenever an abbreviated prostate MRI protocol is being considered for implementation in clinical routine.

## 1. Introduction

Multiparametric prostate MRI (mpMRI) has become an integral part of the clinical assessment in patients with suspected prostate cancer and is used to (a) identify target lesions for subsequent biopsy [1,2], (b) provide information on local tumor staging [3], (c) help in active surveillance approaches [4,5] and (d) is even proposed as a tool for screening in men [6]. The development of the PI-RADS guidelines has further laid the foundation to ensure consistent and reproducible standardized acquisition and reporting, particularly for unexperienced readers [7]. PI-RADS has also contributed to the widespread adoption of mpMRI amongst radiologists and urologists worldwide and is an integral part of both national and international clinical guidelines [8]. Consequently, the number of prostate MRI examinations performed has soared over the last years and is expected to continue to do so [9,10].

However, compared to clinical tests based on laboratory results (e.g., Prostate-Specific Antigen, PSA) or the use of clinical nomograms, mpMRI is considered a time-consuming and comparably costly method. Efforts have therefore been made to reduce the time needed to acquire and interpret prostate MRI (and thus the costs) in order to allow for a larger number of men benefitting from this technique.

This review highlights areas of research and provides clinical expertise on ways to effectively shorten a prostate MRI protocol, including the potential benefits and limitations of each approach. One efficient method to shorten the MRI exam of the prostate is to omit the intravenous injection of contrast media and therefore perform a “biparametric” MRI of the prostate. This method and the question of whether the injection of a contrast agent is still necessary is further discussed in more detail in a separate article in this Special Issue. The authors hope this review can serve as guidance whenever shortened MRI protocols are being implemented in clinical routine.

## 2. Minimum Requirements for Multiparametric Prostate MRI

The current PI-RADS guideline includes detailed technical recommendations on how to perform prostate MRI [7]. Typically, it includes T2-weighted turbo spin-echo sequences in several planes, a diffusion-weighted sequence with acquisitions at several b-values, and a dynamic contrast-enhanced 3D gradient echo sequence with a high temporal resolution. In addition, other sequences (e.g., a T1-weighted sequence to detect hemorrhage after biopsy or bone metastases) are commonly performed.

A prostate MRI examination termed “multiparametric” according to PI-RADS will, at the very least, consist of the following:

- A T2-weighted axial plane and at least one additional T2-weighted sequence in an orthogonal plane (i.e., sagittal or coronal) with a slice thickness of 3 mm (no gap), a Field of View (FoV) of 12–20 cm, and an in-plane resolution ≤0.7 × 0.4 mm;
- A diffusion-weighted sequence with at least one low b-value (preferably 50–100 s/mm²) and one intermediate b-value at 800–1000 s/mm². The “high b-value image” required for analysis (≥1400 s/mm²) can then be separately acquired or calculated from the two lower b-values. The slice thickness should be ≤4 mm (no gap), FoV of 16–22 cm, and an in-plane resolution ≤2.5 × 2.5 mm;
- A 3D T1-weighted gradient echo sequence (slice thickness 3 mm, no gap) with injection of a contrast agent, a temporal resolution ≤15 s, and a total observation time span of ≥2 min.

This approach results in a multiparametric MRI that takes approximately 20–25 min (depending on scanner hardware and the size of the prostate) to acquire and serves as a starting point for further optimizations and abbreviations.

## 3. Areas of Possible Protocol Abbreviations

In addition to omitting helpful, but not mandatory sequences of the full mpMRI protocol (i.e., T1-weighted sequences to assess for hemorrhage or bone metastases, whole-pelvis DWI to identify lymph nodes, etc.), abbreviation is focused on speeding up or removing the “core” T2-weighted, diffusion-weighted, and dynamic contrast-enhanced sequences. Of note, these abbreviated protocols have been implemented to address the typical scenario of a biopsy-naïve patient with suspected prostate cancer. They have not been comprehensively evaluated in other clinical settings, such as high-risk patients with family history of prostate cancer or genetic predispositions, etc., or in patients with prior intervention (biopsies or treatment). Hence, depending on the clinical question asked (e.g., detection, local staging, development under active surveillance), different ways of protocol abbreviations are to be considered.

### 3.1. Use of Dynamic Contrast-Enhanced Sequences

The acquisition of a dynamic contrast-enhanced sequence (DCE) is still preferred for a true multiparametric MRI protocol by the PI-RADS steering committee. However, the importance of DCE in the scoring of prostate lesions is declining since the release of the first PI-RADS guidelines in 2012. In the current PI-RADS version 2.1 from 2019, DCE only plays a minor role in interpretation of suspicious lesions and is commonly regarded as a “backup sequence” if T2-weighted imaging or more often diffusion-weighted imaging is of inferior image quality. Of note, only a few lesions seem to require DCE assessment to be identified. For example, in a large validation study for the PI-RADS 2.0 scoring system, DCE helped detection in only 4 out of 152 tumors [11], and in a study of Kuhl et al. including 542 patients, only one additional clinically significant prostate cancer was found through DCE assessment at the cost of 11 additional false-positive diagnoses [12].

Omitting the injection of a contrast agent and only performing T2-weighted and diffusion-weighted MRI (termed bi-parametric MRI, bpMRI) would obviously shorten examinations times, reduce costs, and free up the time required for IV access, allowing for an increased MR throughput. This increased throughput can result in a substantial cost benefit: Porter et al. calculated the gross profit of performing three bpMRI instead of one mpMRI in a fixed 45 min MRI slot to be more than twice as high [13].

Though gadolinium contrast agents are generally considered safe for clinical use, using a bpMRI protocol would also eliminate the possibility for adverse reactions to the contrast agent [14] and accumulation of gadolinium in bone and brain tissue, the clinical significance of which is yet unknown [15].

Recently, an increasing number of studies have been published that investigated the accuracy of bi-parametric MRI in the detection of target lesions for subsequent biopsies, with the majority of trials indicating a comparable diagnostic performance of bpMRI when compared to the full mpMRI protocol [16,17,18,19,20]. For example, Bosaily et al. conducted a multicenter, multireader trial (PROMIS trial) including 497 biopsy-naïve men undergoing pre-biopsy mpMRI followed by transperineal mapping biopsy as gold standard and found that sensitivity and specificity with and without DCE sequences were virtually identical (94% vs. 95% and 37% vs. 38%, respectively) [19]. Alabousi et al. reported similar results in a meta-analysis of 31 studies, with the area under the receiver operating characteristic curve (ROC) reaching 0.90 for bpMRI and 0.87 for mpMRI [18]. They later verified their results in a prospective cohort [21]. These results align well with the results of a large meta-analysis published by Woo et al., who also reported comparable diagnostic accuracy between mpMRI and bpMRI even when applying subgroup analysis and stratifying by zone (peripheral vs. transition zone), field strength (1.5 vs. 3 T), endorectal vs. phased-array coil, PI-RADS version used, temporal resolution of the DCE sequence, quantitative vs. qualitative DCE assessment, etc. [16]. In a study by Tamada et al. in 103 patients, bpMRI and mpMRI not only showed comparably diagnostic accuracy but also comparable inter-reader agreement [22].

As prostate MRI is not only used to detect target lesions for subsequent biopsy but also plays a role in local staging of the tumor (i.e., in regard to extracapsular extension or seminal vesical invasion), Christophe et al. investigated the difference between bpMRI and mpMRI in assessment of extra-prostatic extension with histopathology after radical prostatectomy as gold standard and found the shorter bpMRI to be equivalent to the complete mpMRI protocol, including DCE sequences [23].

However, preferring bpMRI over mpMRI is not universally accepted, and some authors argued that DCE may be beneficial in patients with indeterminate (PI-RADS score 3) lesions in the peripheral zone and in diagnosing clinically-significant prostate cancer (csPCa) [24,25,26]. In addition, while Xu et al. [27] reported comparable AUC values for bpMRI and mpMRI protocols for both prostate cancer (AUC 0.79 and 0.791) and clinically significant prostate cancer (AUC 0.781 and 0.779), they found positive DCE results to be more common in bpMRI 4 lesions, possibly allowing for an improved stratification of tumor aggressiveness. In addition, there is evidence that DCE is helpful to the less experienced radiologist in non-expert centers: Gatti et al. demonstrated that while expert readers performed well in both bpMRI and mpMRI, inexperienced readers with only 300 cases read performed significantly worse in bpMRI (AUC 0.73 vs. 0.86) [28].

Though DCE sequences may be of limited use in detecting suspicious target lesions for subsequent biopsy (at least in experienced readers), there is evidence that mpMRI may be superior in patients undergoing radical prostatectomy and suspicion for recurrent cancer in the pelvis or evaluation of the prostate for recurrence after high-intensity focused ultrasound (HIFU) treatment [29] although results are controversial. For example, Kitajima et al. reported an improvement in diagnostic accuracy for DCE in patients with suspected local recurrence after radiotherapy, while DWI only showed limited incremental value, possibly due to susceptibility artifacts caused by surgical clips or gas in the rectum [30]. However, Valle et al. and Abd-Alazeez et al. did not see any added value of DCE sequences when assessing possible local recurrence [31,32], and while DCE after HIFU therapy is recommended [29], Lotte et al. did not see a benefit by DCE in their study [33]. For imaging after radiotherapy and in contrast to pre-biopsy MR imaging, the addition of DCE did not improve accuracy for recurrent prostate cancer regardless of the level of experience of the readers [34].

The article by Turkbey et al. in this special issue includes a detailed discussion of the literature on the advantages and disadvantages of mpMRI vs. bpMRI.

The PI-RADS v2.1 guideline currently recommends a total acquisition span of ≥2 min for the DCE sequence. With contrast uptake of a suspicious lesion commonly occurring very early after contrast injection, the full observation time span of 2 min as recommended by the PI-RADS steering committee might not be necessary. Bae et al. investigated the best time cut-off for detecting csPCa and found 60–72 s to be optimal, which would allow for shortening the DCE sequence by an additional 48 s [35].

Ideally, the decision on whether or not to inject contrast (e.g., in cases of degraded image quality on T2-weighted or diffusion-weighted sequences) would be made on-the-fly by a radiologist on a per-case basis. However, this may not be feasible, especially with increasing volumes of prostate MRI examinations being performed at many centers. Hötker et al. [36] trained an artificial intelligence, based on data labeled by expert radiologists, to be able to decide on the necessity for performing a DCE sequence, which allowed them to spare the patient from an unnecessary contrast injection in 48% of patients while falsely omitting DCE in only 2%. Automated software solutions, recommending alterations in acquisition and thus creating patient-tailored multiparametric MRI protocols based on analysis of real-time imaging data and image quality, are certainly a new and interesting field for the application of artificial intelligence.

### 3.2. Shortening T2-Weighted Acquisition Times

In addition to the standard axial T2-weighted sequence, the current PI-RADS guidelines require one additional orthogonal plane in sagittal or coronal orientation. As T2-weighted turbo spin echo (TSE) sequences are one of the most time-consuming elements of the MR protocol, omitting this additional plane would result in time benefits. Combined with the omittance of DCE, this approach, termed “fast bpMRI”, was investigated by Barth et al. and van der Leest at al. [37,38]. The authors used a single-axial T2-weighted sequence in conjunction with a diffusion-weighted sequence in the same orientation to detect PCa in men with elevated PSA levels. The protocol showed a comparable diagnostic accuracy with only a slightly lower specificity for fast bpMRI compared to full bpMRI and mpMRI (0.65 vs. 0.69).

**Figure 1:**
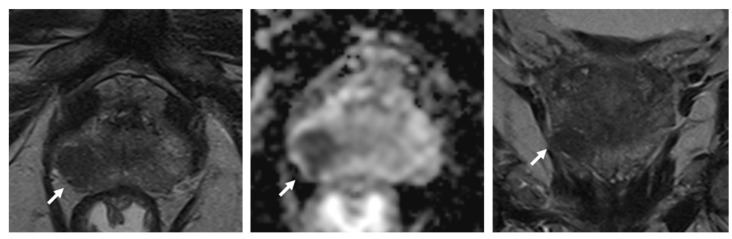
76-year old patient with elevated PSA value and a suspicious lesion (white arrows) in the right peripheral zone. In this case, the extracapsular tumor extension is visualized best on the coronal T2-weighted sequence.

While omitting additional T2-weighted sequences results in substantial time savings, there are potential pitfalls: (1) The evaluation of the transition zone (TZ), according to PI-RADS, heavily relies on T2-weighted images as the predominant series. As features like “well/less circumscribed” and/or “encapsulation” are used to distinguish between tumors and BPH nodules, visualization of the lesion on a different plane may occasionally be helpful. (2) While most studies investigate the use of fast bpMRI/bpMRI in a pre-biopsy or screening setting, prostate MRI is also used to stage tumors regarding extra-prostatic tumor extension including infiltration of the seminal vesicles—an assessment that will likely benefit from additional planes (Figure 1). These disadvantages could obviously be remedied by performing a second, full multiparametric examination only in selected patients, alas at the cost of reducing efficacy.

Recently, new technological improvements have experienced widespread application in MR imaging, most notably simultaneous multi-slice (SMS). This technique allows for the parallel acquisition of several slices at the same time, which can be used to reduce the time needed for a complete multiparametric MRI significantly or to further shorten an already fast bpMRI protocol. As this technique is available for both turbo-spin-echo and DWI sequences, a fast bpMRI protocol including a single-axial T2-weighted and a diffusion-weighted sequence in less than 5 min of combined scan time becomes feasible: Weiss et al. were able to show comparable diagnostic performance in fifty-two patients prospectively undergoing MRI either including a standard DWI sequence or a DWI sequence with SMS and did not find any differences in diagnostic accuracy [39].

Another way to significantly reduce scan times is the use of a 3D TSE sequence instead of separate 2D TSE acquisitions in all planes. If an isotropic 3D acquisition is used, the resultant images could also be reformatted in any plane desired, which might prove beneficial when discriminating between real lesions (in particular those with ill-defined borders) and partial volume effects. Polanec et al. investigated the use of a 3D TSE sequence in comparison to standard 2D TSE sequences and did not find any reported differences in overall image quality, lesion delineation, and diagnostic accuracy [40]. Rosenkrantz et al. and Shankar et al. reported similar results [41,42]. In another study by Caglic et al., the use of a 3D acquisition also seemed to offer increased sensitivity for extracapsular tumor extension [43].

**Figure 2:**
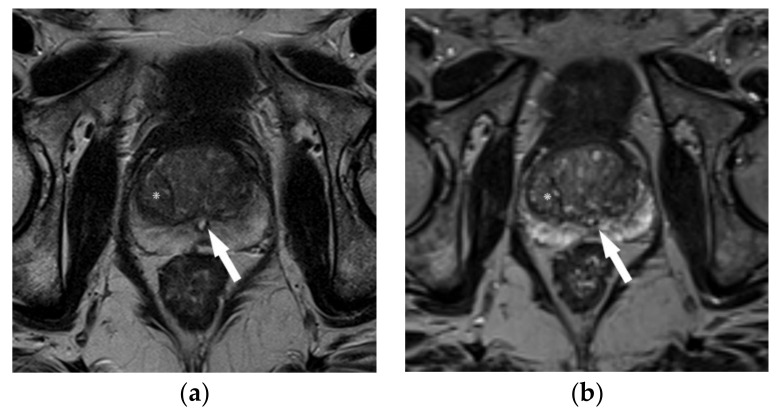
58-year old patient with elevated PSA undergoing MRI before template biopsy. Anatomic landmarks such as the verumontanum (arrow) and BPH-nodule (*) are more clearly depicted on 2D TSE acquisition (**a**) than on isotropic 3D sequences (**b**).

However, 3D acquisitions are currently only recommended as an adjunct to 2D acquisitions in the PI-RADS guideline, as their soft tissue contrast and in-plane resolution may be lower than those of standard 2D sequences [7] (Figure 2).

Finally, new MR techniques based on methods of machine learning have found their way into clinical routine. These new methods are potentially able to reduce noise while also increasing signal to noise ratio and image sharpness and are therefore able to reduce acquisition time. Just recently, deep-learning-accelerated T2-weighted imaging of the prostate was shown to shorten acquisition time from 4.5 min to 1.5 min without sacrificing image quality [44]. Contrarily, subjective analysis of imaging data showed a higher image quality and reduced noise in images postprocessed using a deep learning algorithm. A similar approach to increase image quality using deep learning reconstruction of DWI was recently published by Ueda et al. [45]. DWI at b-values ranging from 1000 to 5000 s/mm^2^ were subjectively and objectively analyzed regarding image quality, which was rated regarding image quality, signal-to-noise ratio, and contrast-to-noise ratio. Both subjective and objective analysis evidenced higher values for the images reconstructed using a deep learning image reconstruction approach. Furthermore, the calculated apparent diffusion coefficient was not influenced by the reconstruction algorithm, therefore guaranteeing validity of this quantitative biomarker even with using deep-learning-based approaches of image reconstruction. However, evaluation of deep-learning-based DWI in a clinical setting was not performed; hence, no statement regarding the performance of deep-learning-based reconstruction in the setting of artifacts, such as hip replacement prosthesis or presence of rectal gas, can be drawn. Further validation of such modern reconstruction algorithms by scientific clinical studies is needed comparing acquisition time, image quality, and diagnostic accuracy of acquisitions with and without machine-learning-based reconstruction algorithms in MRI of the prostate.

### 3.3. Shortening Diffusion-Weighted Imaging Acquisition Times

Obtaining good-quality diffusion-weighted images is pivotal in prostate MRI, particularly when a shortened protocol without DCE and/or only a single-plane T2- weighted sequence is used. As the diffusion-weighted sequence is also commonly the most time-consuming sequence in the prostate MRI protocol, selecting the best sequence and parameters is of importance. While the acquisition of three b-values (e.g., 100, 600, and 1000 s/mm² and a calculated high b-value) is common practice in most institutions, as it allows for a more accurate calculation of the apparent diffusion coefficient (ADC), the PI-RADS only requires at least two b-values (0–50 s/mm² and 800–1000 s/mm²), which reduces acquisition times [7]. The required “high b-value” image (b ≥ 1400 s/mm²) can be calculated from the low and intermediary b-values instead of acquired separately, without compromising detection rates [46].

When applying a bpMRI or fast bpMRI protocol in clinical routine, artifacts on diffusion-weighted images are of concern, as re-calling patients to undergo an additional examination in cases of non-diagnostic image greatly impacts the cost-benefit ratio of abbreviated MR protocols. Unfortunately, the standard single-shot EPI sequence (ssEPI) is susceptible to artifacts caused by air in the adjacent rectum or hip prothesis as well as anatomical distortions induced by magnet field inhomogeneities and eddy currents. In the past, several authors have reported on the use of anti-spasmodic agents or rectal enemas as countermeasures; however, the results are conflicting. Schmidt et al. [47] reported that only application of a microenema prior to the examination improved image quality, a recent meta-analysis did not see that effect but instead recommended intravenous hyoscine butyl-bromide to improve image quality [48]. Technical developments have also been made to improve image quality and reduce artifacts, such as reduced field-of-view with spatially selective excitation pulses DWI sequences (rFoV-DWI) [31,32], read-out-segmented multi-shot EPI DWI sequences [49], or slice-specific shimming [50]; but, while potentially reducing artifacts, these advances usually increase acquisition times.

### 3.4. Optimizing Prostate MRI Workflow

In addition to technical improvements or focusing on selected MR sequences to allow for shortened examination times and improved patient throughput in MRI, several authors investigated ways to increase reporting speed and reduce inter-reader agreement. This is of particular importance, as an increasing number of examinations will most likely not be met with an increased number of radiologists at every center, and a significant delay of the final report should be avoided. Several authors investigated the use of a computer-aided diagnosis (CAD) system [51,52,53,54] and were able to demonstrate significant reductions in reporting times. Different aspects of computer-aided diagnosis and different products were used and described. For example, Gaur et al. conducted a multi-institutional study and found the CAD system to significantly reduce reporting time without sacrificing accuracy [51]. For lesion detection, they used a CAD system developed in-house, which did not consider DCE images, whereas commercially available software was used for automated contouring of the prostate. Giannini et al. and Zhu et al. reported similar results [52,53] using previously validated self-developed software for feature extraction from mpMRI. In addition to CAD as a helpful tool in reporting prostate cancer, applications of artificial intelligence recently have gained a great deal of interest both in supporting the detection of prostate cancer as well as in the effort to reduce reading times. Winkel et al. not only showed a slightly increased detection rate for csPCa when validating a prototype CAD system employing artificial intelligence developed by a commercial vendor but, at the same time, found reduced reading times by 21% [54].

## 4. Conclusions

For the assessment of men with suspected prostate cancer, prostate MRI has become part of the clinical routine, with numerous men benefitting from more precise diagnostics. This new importance of MRI in the clinical management of prostate cancer is consequently accompanied with a steep increase in examination numbers. Several approaches are available to shorten examination times and thus increase throughput for prostate MRI, with omitting the dynamic contrast-enhanced or additional planes on T2-weighted being the most common choices. Though not yet endorsed by the PI-RADS steering committee, there is increasing evidence that these measures would allow for a significant reduction in acquisition times without sacrificing diagnostic accuracy, at least for the experienced reader. Together with technical advances, a combined approach seems to be feasible in patients with suspected prostate cancer or when screening for cancer.
